# Supplementary material for: Evaluation of SARS-CoV-2 entry, inflammation and new therapeutics in human lung tissue cells
Source: PLoS Pathog. 2022 Jan 13;18(1):e1010171. doi: 10.1371/journal.ppat.1010171 (PMC8791477; doi:10.1371/journal.ppat.1010171)
Supplement: S1 Text — (DOCX) [file ppat.1010171.s006.docx]

**Table S1. Antiviral drug candidates for entry inhibition of SARS-CoV-2.**

| **Supplier** | **Catalog #** | **Candidate**  **(generic name)** | **Mechanism of action** | **Current indications and remarkable properties** |
| --- | --- | --- | --- | --- |
|  | | | ***TMPRSS2 inhibitors*** |  |
| Selleckchem | S2874 | **Camostat mesylate** | - Inhibits serine proteases [[[^1^](#_ENREF_1)](#_ENREF_1)](#_ENREF_1). - Prevents TMPRSS2 to activate SARS-CoV-2 spike protein for viral entry ^[2](#_ENREF_2)^. | - Antiproteinuric drug [^1^](#_ENREF_1). - Approved for pancreatitis in Japan ^[2](#_ENREF_2)^. |
|  | | | ***Corticosteroids*** |  |
|  | | | - Block the initial inflammatory response by inhibiting the production of nitric oxide and eicosanoids that promote vascular permeability and vasodilation. As a result, leukocyte migration to inflamed tissues is decreased [^3^](#_ENREF_3). - Modulate events in chronic inflammation by altering/suppressing T cell activation [^3^](#_ENREF_3). | - Immune-mediated diseases, e.g., rheumatoid arthritis, allergies, ulcerative colitis, lupus erythematosus and skin disorders such as psoriasis and dermatitis [^3^](#_ENREF_3). |
| Selleckchem | S4430 | **Cortisol** | - Major glucocorticoid in humans [^3^](#_ENREF_3). | - It has disadvantageous salt retaining properties which distinguishes it from other glucocorticoids [^4^](#_ENREF_4). |
| Sigma-Aldrich | D1756 | **Dexamethasone** | - Possesses 20 to 30 times the binding affinity for glucocorticoid receptors of endogenous cortisol [^5^](#_ENREF_5). | - Treatment for post-operative and chemotherapy-induced nausea and vomiting [^5^](#_ENREF_5). |
| Selleckchem | S2121 | **Ciclesonide** | - Converted in the lower respiratory tract to an active metabolite with 100-fold greater relative glucocorticoid receptor binding affinity than ciclesonide itself [^6^](#_ENREF_6) [^7^](#_ENREF_7). | - Asthma prevention [^7^](#_ENREF_7). |
| Selleckchem | S1696 | **Prednisone** | - Metabolized in the liver to its active metabolite [^8^](#_ENREF_8). | - First choice in miastenia gravis [^8^](#_ENREF_8). - Maintenance therapy for kidney transplant patients [^8^](#_ENREF_8). |
|  | | | ***Nonsteroidal anti-inflammatory drugs (NSAIDs)*** |  |
|  |  |  | - Inhibit ciclooxygenases (COXs), causing a reduction in the production of prostaglandins at the site of tissue injury and attenuation of the inflammatory cascade [^9^](#_ENREF_9). | - Provide analgesia for mild to moderate pain resulting from surgery, injury, and disease [^9^](#_ENREF_9). - Anti-inflammatory properties [^9^](#_ENREF_9). |
| Selleckchem | S1261 | **Ibuprofen** | - Inhibits COX [^9^](#_ENREF_9). - Exerts a direct spinal action by blocking the hyperalgesic response induced by activation of spinal glutamate and substance P receptors [^9^](#_ENREF_9). | - Widely used as an analgesic, anti-inflammatory, and antipyretic [^10^](#_ENREF_10). |
| Selleckchem | S1622 | **Licofelone** | - Inhibits COX and lipoxigenase (LOX) [^11^](#_ENREF_11). | - Phase III trials have been successfully completed as a treatment for osteoarthritis[^11^](#_ENREF_11). |
| Selleckchem | S1638 | **Sulindac** | - Converted *in vivo* to an active sulfide compound by liver enzymes [^12^](#_ENREF_12). - Inhibits COX [^12^](#_ENREF_12). - Modulates Wnt/β-catenin as well as NF-κB signalling that can have repercussion on the invasive behaviour of cancer cells [^13^](#_ENREF_13). | - Relief of signs and symptoms of several arthritic conditions [^12^](#_ENREF_12). - Induction of cholestatic liver injury due to its competitive inhibition of canalicular bile acid transport [^14^](#_ENREF_14). |
| Selleckchem | S2386 | **Celecoxib** | - Selectively inhibits COX-2 [^15^](#_ENREF_15). | - Pain relief in osteoarthritis [^15^](#_ENREF_15). - Reduction of precancerous polyps in the colon [^16^](#_ENREF_16). - Potentiation of the anticoagulant effects of warfarin. Serious bleeding complications have been reported [^17^](#_ENREF_17). |
|  | | | ***Other immunomodulators*** |  |
| Selleckchem | S4238 | **Cepharanthine** | - Alkaloid isolated from *Stephania cepharantha* [^18^](#_ENREF_18). - Modulates efflux pumps and membrane rigidification [^19^](#_ENREF_19). - Reduces inflammation by AMPK activation and NF-κB inhibition [^19^](#_ENREF_19). - Inhibits plasma membrane lipid peroxidation and platelet aggregation [^20^](#_ENREF_20). - Suppresses cytokine production [^20^](#_ENREF_20). | - Used in Japan since the 1950s to treat leukopenia, snake bites, xerostomia and alopecia areata [^19^](#_ENREF_19). - Immunoregulatory, anti-oxidative, -inflammatory, -cancer, -viral and -parasitic properties [^19^](#_ENREF_19). |
| Selleckchem | S4008 | **Pemirolast potassium** | - Stabilizes mast cells [^21^](#_ENREF_21). | - Prevention and relief of ocular manifestations of allergic conjunctivitis [^21^](#_ENREF_21). |
|  | | | ***For enzymatic deficiencies*** |  |
| Selleckchem | S4680 | **Protilerin** | - Stimulates the release of thyroid-stimulating hormone from the anterior pituitary gland [^22^](#_ENREF_22). | - Stimulation test to diagnose hyperthyroidism [^23^](#_ENREF_23). |
| Sigma-Aldrich | T4425 | **Tetrahydrobiopterin dihydrochloride** | - Essential cofactor required for the synthesis of several neurotransmitters [^24^](#_ENREF_24). | - Tetrahydrobiopterin deficiency (phenylketonuria) [^25^](#_ENREF_25). |
|  | | | ***Blood regulation*** |  |
| Selleckchem | S5084 | **Carbazochrome sodium sulfonate** | - Reduces capillary permeability [^26^](#_ENREF_26). - Hemostatic agent that promotes clotting [^27^](#_ENREF_27). | - Pain relief in refractory chronic prostatitis [^26^](#_ENREF_26). - In combination with tranexamic acid for the reduction of perioperative blood loss and inflammatory response [^28^](#_ENREF_28). - In combination with vitamin C, vitamin E and lysozyme for the reduction of gingival inflammation in chronic periodontitis [^29^](#_ENREF_29). - In combination with procaine results in better efficacy and less adverse effects in treating moderate to massive hemoptysis than vasopressin [^30^](#_ENREF_30). |
| MedChemExpress | HY-B0799 | **Ergoloid mesylates** | - Binds with high affinity to the γ-aminobutyric acid (GABA)_A_ receptor Cl^-^ channel, producing an allosteric interaction with the benzodiazepine site [^31^](#_ENREF_31). - Dihydrogenation eliminates vasoconstrictor effects of ergotoxine and enhances its α-adrenoreceptor and 5-hydroxytryptamine (serotonin) receptor antagonist properties [^32^](#_ENREF_32). - Inhibits brain-specific phosphodiesterases [^32^](#_ENREF_32). | - Supposed therapeutic effects in depression, confusion, lack of self-care in the elderly, and erectile dysfunction [^32^](#_ENREF_32). |
| Sigma-Aldrich | SML2313 | **Higenamine hydrochloride** | - Alkaloid found in *Aconitum* plant [^33^](#_ENREF_33). - Reduces IL-1β-induced inflammation in human nucleus pulposus cells via inhibiting NF-κB signaling pathway [^34^](#_ENREF_34). - Protects neuronal cells against oxygen-glucose deprivation/reperfusion (OGD/R)-induced injury by regulating the Akt and Nrf2/HO-1 signaling pathways [^35^](#_ENREF_35). - Possesses positive ionotropic and chronotropic, activating slow channel, vascular and tracheal relaxation effects [^33^](#_ENREF_33). | - Collapse, syncope, painful joints, edema, and bronchial asthma treatment in Asian traditional medicine [^33^](#_ENREF_33). - Antispasmodic for Raynaud's phenomenon and cold-induced vasoconstriction [^36^](#_ENREF_36). - Potential therapeutic effects for diseases like intravertebral disc degeneration [^34^](#_ENREF_34), heart failure, disseminated intravascular coagulation, ischemia/reperfusion injuries and erectile dysfunction [^33^](#_ENREF_33). - Immunomodulatory, anti-inflammatory, -thrombotic, -apoptotic and -oxidative properties [^33^](#_ENREF_33). |

|  | | | ***Antidiabetics*** |  |
| --- | --- | --- | --- | --- |
| Vall d’Hebron Pharmacy Department |  | **Metformin** | - Reduces gluconeogenesis and hepatic glucose production and improves insulin sensitivity by increasing peripheral glucose uptake [^37^](#_ENREF_37). - Increases anaerobic glucose metabolism in enterocytes [^38^](#_ENREF_38). - Accumulates in the mitochondria to inhibit mitochondrial complex I, leading to increased cytoplasmic ADP:ATP and AMP:ATP ratios. These changes activate AMPK to regulate glucose metabolism [^38^](#_ENREF_38). | - First choice drug for Type 2 diabetes [^39^](#_ENREF_39). |
| Selleckchem | S2542 | **Phenformin** | - Acts on the cell membrane to decrease oxidative phosphorylation [^40^](#_ENREF_40). - Produces tissue anoxia [^40^](#_ENREF_40). - Increases peripheral glucose uptake (Pasteur Effect) [^40^](#_ENREF_40). - Leads to lactic acidosis by inhibition of lactic acid metabolism [^40^](#_ENREF_40). | - Predecessor of metformin with an unacceptably high incidence of lactic acidosis, often fatal [^41^](#_ENREF_41). |
| Vall d’Hebron Pharmacy Department |  | **Vildagliptin** | - Selectively inhibits dipeptidyl peptidase-4 (DPP-4), an enzyme that degrades and inactivates glucagon-like peptide-1 (GLP-1) and glucose-dependent insulinotropic polypeptide (GIP), which promote insulin secretion and regulate blood glucose levels [^42^](#_ENREF_42). | - Type 2 diabetes [^43^](#_ENREF_43). |
| Vall d’Hebron Pharmacy Department |  | **Sitagliptin** | - Selectively inhibits DPP-4 [^43^](#_ENREF_43). - Elevates GLP-1 levels to increase insulin release after meals and improve glucose tolerance [^43^](#_ENREF_43). | - Type 2 diabetes [^43^](#_ENREF_43). |

|  | | | ***Natural compounds - Flavonoids*** |  |
| --- | --- | --- | --- | --- |
|  | | | - Possess several anticancer effects: they modulate reactive oxygen species (ROS)-scavenging enzyme activities, participate in arresting the cell cycle, induce apoptosis, autophagy, and suppress cancer cell proliferation and invasiveness [^44^](#_ENREF_44). | - Multiple potential applications. |
| Selleckchem | S2320 | **Luteolin** | - Found in a number of dietary sources [^45^](#_ENREF_45). | - Plants rich in luteolin have been used in Chinese traditional medicine for hypertension, inflammatory disorders, and cancer treatment [^46^](#_ENREF_46). |
| Sigma-Aldrich | 94258 | **Eriodictyol** | - Present in citrus fruits and Chinese herbs used in the food industry [^47^](#_ENREF_47). - Reduces inflammation by NF-κB blockade [^48^](#_ENREF_48). - As opposite to other flavonoids, eriodictyol lacks the C2–C3 double bond responsible to give more inhibitory activity of basal vascular NO release and vascular superoxide formation [^49^](#_ENREF_49) | - Anti-inflammatory, -allergenic, -microbial, -cancer, and -oxidant properties [^48^](#_ENREF_48). |
| Selleckchem | S2391 | **Quercetin** | - Obtained from diverse fruits and vegetables [^50^](#_ENREF_50). - Stabilizes basophils and mast cells [^50^](#_ENREF_50). - Reduces obesity by increase in AMPK expression [^51^](#_ENREF_51). - Inhibits histone deacetylase 1 and DNA methyltransferase 1 [^51^](#_ENREF_51). | - Antioxidative, -inflammatory, -platelet, -apoptotic, -invasive and -angiogenic properties [^51^](#_ENREF_51). - Nephro-, gastro-, angio-, cardio- and chondroprotective properties [^52^](#_ENREF_52). |
| Selleckchem | S2007 | **Myricetin** | - Found in tea, berries, fruits, vegetables, and the plant *Diospyros lotus* [^53^](#_ENREF_53). - Inhibits thrombin with an IC_50_ value of 56 μM [^54^](#_ENREF_54). - Inhibits the production of proinflammatory mediators through the suppression of NF-κB and STAT1 activation and induction of Nrf2-mediated HO-1 expression in LPS-stimulated RAW264.7 macrophages [^55^](#_ENREF_55). | - *Diospyros lotus* is traditionally used in diabetes, diarrhea, tumor, and hypertension treatment [^55^](#_ENREF_55). |

|  | | | ***Natural compounds - Others*** |  |
| --- | --- | --- | --- | --- |
| Selleckchem | S2326 | **Glycyrrhizin** | - Triterpenoid saponin found as the major active constituent in licorice root [^56^](#_ENREF_56). - Shows anti-inflammatory and antioxidant activities [^57^](#_ENREF_57). - Stimulates endogenous production of interferons [^57^](#_ENREF_57). - Induces melanogenesis through cAMP signaling [^58^](#_ENREF_58). | - Bronchitis, gastritis, and jaundice alleviation in traditional medicine [^56^](#_ENREF_56). |
| Selleckchem | S4646 | **Indirubin** | - Found in a variety of plants, marine mollusks, bacteria, and human urine, and produced in mammals’ intestine [^59^](#_ENREF_59). - Inhibits several cyclin-dependent kinases and glycogen synthase kinase 3 [^59^](#_ENREF_59). - May suppress lipopolysaccharides-induced inflammation via Toll-like receptor 4 abrogation mediated by the NF-κB and MAPK signaling pathways [^60^](#_ENREF_60). | - Chronic myelogenous leukemia treatment in Chinese traditional medicine [^59^](#_ENREF_59). - Indirubin in Lindioil ointment for psoriasis topical treatment [^61^](#_ENREF_61). |
| MedChemExpress | HY-N0453 | **Hypericin** | - Produces primary photosensitization [^62^](#_ENREF_62). - Shows selective activity against viruses, both *in vitro* and *in vivo*, including Herpes simplex (HSV)-1 and -2 [^63^](#_ENREF_63). | - Non-melanoma skin cancer topical treatment [^62^](#_ENREF_62). |
|  | | | ***Antimicrobials*** |  |
| Sigma-Aldrich | 90527 | **Hydroxychloroquine** | - Inhibits pH-dependent viral fusion/replication [^1^](#_ENREF_1). - Prevents viral envelope glycoprotein as well as host receptor protein glycosylation [^1^](#_ENREF_1). - Prevents virion assembly in endoplasmic reticulum-Golgi intermediate compartment–like structure [^1^](#_ENREF_1). - Inhibits TLR-7/9–dependent inflammatory responses [^1^](#_ENREF_1). | - Malaria [^64^](#_ENREF_64). - Rheumatoid arthritis [^64^](#_ENREF_64). - Systemic lupus erythematosus [^64^](#_ENREF_64). |
| Sigma-Aldrich | M2140 | **Monocaprin** | - Safe functional emulsifier in food industry [^65^](#_ENREF_65). - May be a potential preservative independent of pH, acting by disruption of the cell wall and plasma membrane of fungi [^65^](#_ENREF_65). | - Hydrogel formulations have *in vitro* microbicidal activity against HIV and HSV, *Chlamydia trachomatis* and *Neisseria gonorrhoeae* [^66^](#_ENREF_66). - In combination with doxycycline as a potential treatment of herpes labialis [^67^](#_ENREF_67). |
| Sigma-Aldrich | I8898 | **Ivermectin** | - GABA receptor agonist [^68^](#_ENREF_68). - Exerts toxicity in parasites by blocking the post-synaptic transmission of nerve impulses [^68^](#_ENREF_68). | - Broad-spectrum anti-parasite medication [^69^](#_ENREF_69). - First choice treatment for onchocerciasis [^69^](#_ENREF_69). |
| Vall d’Hebron Pharmacy Department |  | **Nitrofurantoin** | - Bacterial intracellular nitroreductases produce the active form of the drug [^70^](#_ENREF_70). - Intermediate metabolites bind to bacterial ribosomes and inhibit bacterial enzymes involved in the synthesis of DNA, RNA, and bacterial wall protein synthesis [^70^](#_ENREF_70). | - Uncomplicated urinary tract infections resistant to other antibiotics [^71^](#_ENREF_71). |
| Fisher Scientific | 10387340 | **Lauric acid** | - Found in vegetal oils [^72^](#_ENREF_72). - Disrupts the cell membrane of gram-positive bacteria by physicochemical processes [^72^](#_ENREF_72). - Interferes with bacterial cell signal transduction and gene transcription processes [^72^](#_ENREF_72). - Activates TLR4 signaling [^73^](#_ENREF_73). | - Main antiviral and antibacterial substance found in human breast milk [^74^](#_ENREF_74). |

| Selleckchem | S3132 | **Sulfamethoxazole** | - A sulfonamide derivative. Sulfonamides have a bacteriostatic effect by inhibiting bacterial folic acid synthesis [^75^](#_ENREF_75). | - In combination with trimethoprim for urinary tract infections, otitis media, chronic bronchitis, *Shigella* and enterotoxigenic *Escherichia coli* infections [^76^](#_ENREF_76). - Prophylaxis of *Pneumocystis jirovecii* pneumonia in patients with HIV/AIDS [^77^](#_ENREF_77). - High rates of adverse drug reactions in subjects infected by HIV [^78^](#_ENREF_78). |
| --- | --- | --- | --- | --- |
| Selleckchem | S2302 | **Sulfamerazine** | - A sulfonamide derivative [^75^](#_ENREF_75). | - Bronchitis, prostatitis, and urinary tract infections [^79^](#_ENREF_79). - Combined with a folate antagonist, sulfonamides are indicated among others in toxoplasmosis and malaria [^80^](#_ENREF_80). |
| Vall d’Hebron Pharmacy Department |  | **Tazobactam** | - Class A β-lactamase inhibitor [^81^](#_ENREF_81). | - Extension of antibiotic’s spectrum of activity [^81^](#_ENREF_81). - Increase in β-lactamic antibiotic stability against bacterial β-lactamases [^81^](#_ENREF_81). |
| Selleckchem | S1784 | **Vidarabine** | - Purine nucleoside analogue [^80^](#_ENREF_80). - Competitively inhibits DNA-dependent DNA polymerases of some DNA viruses approximately 40 times more than those of host cells [^80^](#_ENREF_80). | - The degree of maximal resistance to vidarabine is 4-fold, much lower than the 100-fold resistance to acyclovir with similar DNA-polymerase resistant mutations [^80^](#_ENREF_80). - No longer used due to toxicity issues as well as the discovery of more potent and safer compounds such as acyclovir, which today is widely prescribed for HSV [^82^](#_ENREF_82). |
| Sigma-Aldrich | M1765 | **Monolaurin** | - Solubilizes the lipids and phospholipids in the envelope of pathogenic viruses and bacteria causing the disintegration of their envelopes [^74^](#_ENREF_74). | - Inactivation to some extent of HIV, measles, HSV-1, vesicular stomatitis, Visna virus, and cytomegalovirus [^74^](#_ENREF_74). |
| Sigma-Aldrich | PHR1949 | **Sodium lauryl sulfate** | - Ionic detergent that rapidly disrupts biological membranes [^83^](#_ENREF_83). | - Inhibition of the formation of several bacterial biofilms [^84^](#_ENREF_84). |
| Selleckchem | S1915 | **Valaciclovir** | - Purine nucleoside analogue [^80^](#_ENREF_80). - Rapidly and extensively converted to aciclovir by first-pass metabolism [^85^](#_ENREF_85). | - Highly active against Herpes simplex and Herpes zoster [^85^](#_ENREF_85). |

**REFERENCES**

1. Hosoki K, Chakraborty A, Sur S. Molecular mechanisms and epidemiology of COVID-19 from an allergist’s perspective. *Journal of Allergy and Clinical Immunology.* 2020;146(2):285-299.

2. Hoffmann M, Hofmann-Winkler H, Smith JC, et al. Camostat mesylate inhibits SARS-CoV-2 activation by TMPRSS2-related proteases and its metabolite GBPA exerts antiviral activity. *EBioMedicine.* Mar 2021;65:103255.

3. Cruz-Topete D, Cidlowski JA. Glucocorticoids: Molecular Mechanisms of Action. In: Riccardi C, Levi-Schaffer F, Tiligada E, eds. *Immunopharmacology and Inflammation*. Cham: Springer International Publishing; 2018:249-266.

4. Hindmarsh PC, Geertsma K. Chapter 20 - Hydrocortisone. In: Hindmarsh PC, Geertsma K, eds: Academic Press; 2017:231-249.

5. Zabirowicz ES, Gan TJ. 34 - Pharmacology of Postoperative Nausea and Vomiting. In: Hemmings HC, Egan TD, eds. Second Edition ed. Philadelphia: Elsevier; 2019:671-692.

6. Ciclesonide. In: Aronson JK, ed. *Meyler's Side Effects of Drugs*. Sixteenth Edition ed. Oxford: Elsevier; 2016:292-294.

7. Kercsmar CM, McDowell KM. 45 - Wheezing in Older Children: Asthma. In: Wilmott RW, Deterding R, Li A, et al., eds. Ninth Edition ed. Philadelphia: Elsevier; 2019:686-721.e684.

8. Silvestri NJ, Barohn RJ, Wolfe GI. 145 - Acquired Disorders of the Neuromuscular Junction. In: Swaiman KF, Ashwal S, Ferriero DM, et al., eds. Sixth Edition ed: Elsevier; 2017:1098-1105.

9. Walker BJ, Polaner DM, Berde CB. 44 - Acute Pain. In: Coté CJ, Lerman J, Anderson BJ, eds. Sixth Edition ed. Philadelphia: Elsevier; 2019:1023-1062.e1015.

10. DrugBank. Ibuprofen. https://go.drugbank.com/drugs/DB01050.

11. Information NCfB. PubChem Compound Summary for CID 133021, Licofelone. 2021.

12. DrugBank. Sulindac. https://go.drugbank.com/drugs/DB00605.

13. Sherbet GV. Chapter 25 - S100A4 Has Potential Benefits as a Therapeutic Target. In: Sherbet GV, ed: Academic Press; 2017:211-221.

14. Manautou JE, Campion SN, Aleksunes LM. 9.08 - Regulation of Hepatobiliary Transporters during Liver Injury. In: McQueen CA, ed. Second Edition ed. Oxford: Elsevier; 2010:175-220.

15. Roy J. 11 - Life-style drugs, statins, and COX-2 drugs. In: Roy J, ed: Woodhead Publishing; 2011:297-325.

16. DrugBank. Celecoxib. https://go.drugbank.com/drugs/DB00482.

17. Celecoxib. In: Aronson JK, ed. *Meyler's Side Effects of Drugs*. Sixteenth Edition ed. Oxford: Elsevier; 2016:191-195.

18. Nair A, Chattopadhyay D, Saha B. Chapter 17 - Plant-Derived Immunomodulators. In: Ahmad Khan MS, Ahmad I, Chattopadhyay D, eds: Academic Press; 2019:435-499.

19. Bailly C. Cepharanthine: An update of its mode of action, pharmacological properties and medical applications. *Phytomedicine.* 2019;62:152956-152956.

20. Rogosnitzky M, Danks R. Therapeutic potential of the biscoclaurine alkaloid, cepharanthine, for a range of clinical conditions. *Pharmacological Reports.* 2011;63(2):337-347.

21. Bielory L, Bielory BP, Wagner RS. 54 - Allergic and Immunologic Eye Disease. In: Leung DYM, Szefler SJ, Bonilla FA, Akdis CA, Sampson HA, eds. Third Edition ed. London: Elsevier; 2016:482-497.e483.

22. Pirahanchi Y, Toro F, Jialal I. Physiology, Thyroid Stimulating Hormone. 2020; https://[www.ncbi.nlm.nih.gov/books/NBK499850/](http://www.ncbi.nlm.nih.gov/books/NBK499850/).

23. Manolov D, Zakharieva B, Lazarov G, Manov A, Vŭrbanov V. The TRH test using the Bulgarian preparation Protilerin in the diagnosis of hyperthyroidism. *Vutreshni bolesti.* 1989;28(5):30-33.

24. Bhagavan NV, Ha C-E. Chapter 15 - Protein and Amino Acid Metabolism. In: Bhagavan NV, Ha C-E, eds. *Essentials of Medical Biochemistry*. Second Edition ed. San Diego: Academic Press; 2015:227-268.

25. Muntau AC, Adams DJ, Bélanger-Quintana A, et al. International best practice for the evaluation of responsiveness to sapropterin dihydrochloride in patients with phenylketonuria. *Molecular Genetics and Metabolism.* 2019;127(1):1-11.

26. Oh-oka H, Yamada T, Noto H, et al. Effect of carbazochrome sodium sulfonate on refractory chronic prostatitis. *International Journal of Urology.* 2014;21(11):1162-1166.

27. DrugBank. Carbazochrome. https://go.drugbank.com/drugs/DB09012.

28. Luo Y, Zhao X, Releken Y, Yang Z, Pei F, Kang P. Hemostatic and Anti-Inflammatory Effects of Carbazochrome Sodium Sulfonate in Patients Undergoing Total Knee Arthroplasty: A Randomized Controlled Trial. *The Journal of Arthroplasty.* 2020;35(1):61-68.

29. Hong J-Y, Lee J-S, Choi S-H, et al. A randomized, double-blind, placebo-controlled multicenter study for evaluating the effects of fixed-dose combinations of vitamin C, vitamin E, lysozyme, and carbazochrome on gingival inflammation in chronic periodontitis patients. *BMC Oral Health.* 2019;19(40).

30. Ren CS, Du YP, Yu XY. The effect of Procaine combined with Carbazochrome on moderate to massive hemoptysis due to tuberculosis. *Med. J. West China.* 2011;23:1045-1046.

31. MedChemExpress. Dihydroergotoxine mesylate. https://[www.medchemexpress.com/Dihydroergotoxine_mesylate.html](http://www.medchemexpress.com/Dihydroergotoxine_mesylate.html).

32. Ergot derivatives. In: Aronson JK, ed. *Meyler's Side Effects of Drugs*. Sixteenth Edition ed. Oxford: Elsevier; 2016:86-92.

33. Zhang N, Lian Z, Peng X, Li Z, Zhu H. Applications of Higenamine in pharmacology and medicine. *Journal of Ethnopharmacology.* 2017;196:242-252.

34. Bai X, Ding W, Yang S, Guo X. Higenamine inhibits IL-1β-induced inflammation in human nucleus pulposus cells. *Bioscience Reports.* 2019;39(6).

35. Zhang Y, Zhang J, Wu C, et al. Higenamine protects neuronal cells from oxygen‐glucose deprivation/reoxygenation‐induced injury. *Journal of Cellular Biochemistry.* 2019;120(3):3757-3764.

36. Guan J, Lin H, Xie M, et al. Higenamine exerts an antispasmodic effect on cold‑induced vasoconstriction by regulating the PI3K/Akt, ROS/α2C‑AR and PTK9 pathways independently of the AMPK/eNOS/NO axis. *Experimental and Therapeutic Medicine.* 2019;18(2):1299-1308.

37. Day C. Chapter 26 - New Therapies in Obesity. In: Weaver JU, ed: Elsevier; 2018:271-279.

38. DrugBank. Metformin. https://go.drugbank.com/drugs/DB00331.

39. Brindle PK. Chapter 253 - Transcriptional Regulation via the cAMP Responsive Activator CREB. In: Bradshaw RA, Dennis EA, eds. Second Edition ed. San Diego: Academic Press; 2010:2077-2081.

40. Vaman Rao C. Biguanides. In: Wexler P, ed. Second Edition ed. New York: Elsevier; 2005:271-272.

41. DrugBank. Phenformin. https://go.drugbank.com/drugs/DB00914.

42. Sonia TA, Sharma CP. 1 - Diabetes mellitus – an overview. In: Sonia TA, Sharma CP, eds: Woodhead Publishing; 2014:1-57.

43. Bennett RG. Sitagliptin. *Reference Module in Biomedical Sciences*: Elsevier; 2018.

44. Kopustinskiene DM, Jakstas V, Savickas A, Bernatoniene J. Flavonoids as anticancer agents. *Nutrients.* 2020;12(2):457-457.

45. Omar SH. Chapter 4 - Biophenols: Impacts and Prospects in Anti-Alzheimer Drug Discovery. In: Brahmachari G, ed. *Discovery and Development of Neuroprotective Agents from Natural Products*: Elsevier; 2018:103-148.

46. Avendaño C, Menéndez JC. Cancer Chemoprevention. *Medicinal Chemistry of Anticancer Drugs*. Second Edition ed: Elsevier; 2015:701-723.

47. Rajan VK, Muraleedharan K, Hussan KPS. Structural Evaluation and Toxicological Study of a Bitter Masking Bioactive Flavanone, ‘Eriodictyol’. *Polyphenols: Prevention and Treatment of Human Disease*. Second Edition ed: Elsevier; 2018:45-60.

48. Shukla R, Pandey V, Vadnere GP, Lodhi S. Chapter 18 - Role of Flavonoids in Management of Inflammatory Disorders. In: Watson RR, Preedy VR, eds. *Bioactive Food as Dietary Interventions for Arthritis and Related Inflammatory Diseases*. Second Edition ed: Academic Press; 2019:293-322.

49. Ribeiro D, Fernandes E, Freitas M. Flavonoids as Modulators of Neutrophils' Oxidative Burst: Structure-Activity Relationship. Elsevier; 2018:261-276.

50. Horwitz RJ. The Allergic Patient. Elsevier; 2018:300-309.e302.

51. Woon ECY, Toh JDW. Chapter 6 - Antiobesity Effects of Natural Products from an Epigenetic Perspective. *Studies in Natural Products Chemistry.* Vol 41, 2014:161-193.

52. Shebeko SK, Zupanets IA, Popov OS, Tarasenko OO, Shalamay AS. Effects of Quercetin and Its Combinations on Health. Elsevier; 2018:373-394.

53. Jadeja RN, Devkar RV. Polyphenols and Flavonoids in Controlling Non-Alcoholic Steatohepatitis. Elsevier; 2014:615-623.

54. Wang X, Yang Z, Su F, et al. Study on structure activity relationship of natural Flavonoids against thrombin by molecular docking virtual screening combined with activity evaluation *in vitro*. *Molecules.* 2020;25(2):422-422.

55. Cho BO, Yin HH, Park SH, Byun EB, Ha HY, Jang SI. Anti-inflammatory activity of myricetin from *Diospyros lotus* through suppression of NF-κB and STAT1 activation and Nrf2-mediated HO-1 induction in lipopolysaccharide-stimulated RAW264.7 macrophages. *Bioscience, Biotechnology, and Biochemistry.* 2016;80(8):1520-1530.

56. Girish C, Pradhan SC. Herbal Drugs on the Liver. Elsevier; 2017:605-620.

57. Ramos-Tovar E, Muriel P. Phytotherapy for the Liver. In: Ronald Ross Watson VRP, ed. *Dietary Interventions in Liver Disease*: Academic Press; 2019:101-121.

58. Blom van Staden A, Lall N. Chapter 5 - Medicinal Plants as Alternative Treatments for Progressive Macular Hypomelanosis. *Medicinal Plants for Holistic Health and Well-Being*: Elsevier; 2018:145-182.

59. Cragg GM, Newman DJ. Natural Product Sources of Drugs: Plants, Microbes, Marine Organisms, and Animals. Elsevier; 2007:355-403.

60. Lai J-l, Liu Y-h, Liu C, et al. Indirubin inhibits LPS-induced inflammation via TLR4 abrogation mediated by the NF-kB and MAPK signaling pathways. *Inflammation.* 2017;40(1):1-12.

61. Lin YK, See LC, Huang YH, Chi CC, Hui RCY. Comparison of indirubin concentrations in indigo naturalis ointment for psoriasis treatment: a randomized, double‐blind, dosage‐controlled trial. *British Journal of Dermatology.* 2018;178(1):124-131.

62. Penjweini R, Deville S, Ethirajan A, Ameloot M. Chapter 22 - Investigating the Intracellular Dynamics of Hypericin-Loaded Nanoparticles and Polyvinylpyrrolidone-Hypericin by Image Correlation Spectroscopy. In: Hamblin MR, Avci P, Prow TW, eds. Boston: Academic Press; 2016:275-286.

63. Romm A, Clare B, Alschuler L, Hobbs C, Upton R. Chapter 8 - Vaginal Infections and Sexually Transmitted Diseases. In: Romm A, Hardy ML, Mills S, eds. Saint Louis: Churchill Livingstone; 2010:256-289.

64. Howard B. Hydroxychloroquine. In: S.J. Enna DBB, ed. *xPharm: The Comprehensive Pharmacology Reference*: Elsevier; 2007:1-4.

65. Ma M, Wen X, Xie Y, et al. Antifungal activity and mechanism of monocaprin against food spoilage fungi. *Food Control.* 2018;84:561-568.

66. Neyts J, Kristmundsdottir T, De Clercq E, Thormar H. Hydrogels containing monocaprin prevent intravaginal and intracutaneous infections with HSV-2 in mice: Impact on the search for vaginal microbicides. *Journal of Medical Virology.* 2000;61(1):107-110.

67. Skulason S, Holbrook WP, Thormar H, Gunnarsson GB, Kristmundsdottir T. A study of the clinical activity of a gel combining monocaprin and doxycycline: a novel treatment for herpes labialis. *Journal of Oral Pathology & Medicine.* 2012;41(1):61-67.

68. Gupta RC, Milatovic D. Chapter 23 - Insecticides. In: Gupta RC, ed. *Biomarkers in Toxicology*. Boston: Academic Press; 2014:389-407.

69. Bogitsh BJ, Carter CE, Oeltmann TN. Chapter 17 - Blood and Tissue Nematodes. In: Burton J. Bogitsh CEC, Thomas N. Oeltmann, ed. *Human Parasitology*. Fifth Edition ed: Academic Press; 2019:313-329.

70. Squadrito FJ, del Portal D. Nitrofurantoin. *StatPearls*. Treasure Island (FL)2021.

71. Howard B, Furman B. Nitrofurantoin. *Reference Module in Biomedical Sciences*: Elsevier; 2018.

72. Dayrit FM. The properties of lauric acid and their significance in coconut oil. *Journal of the American Oil Chemists' Society.* 2015;92(1):1-15.

73. Wang L, Luo L, Zhao W, et al. Lauric Acid Accelerates Glycolytic Muscle Fiber Formation through TLR4 Signaling. *Journal of agricultural and food chemistry.* 2018;66(25):6308-6316.

74. Lieberman S, Enig MG, Preuss HG. A review of monolaurin and lauric acid: natural virucidal and bactericidal agents. *Alternative and Complementary Therapies.* 2006;12(6):310-314.

75. Singh AK, Sharma AK, Khan I, Gothwal A, Gupta L, Gupta U. Chapter 9 - Oral drug delivery potential of dendrimers. In: Andronescu E, Grumezescu AM, eds. *Nanostructures for Oral Medicine*: Elsevier; 2017:231-261.

76. DrugBank. Sulfamethoxazole. https://go.drugbank.com/drugs/DB01015.

77. Walker L, Yip V, Pirmohamed M. Chapter 20 - Adverse Drug Reactions. In: Padmanabhan S, ed. San Diego: Academic Press; 2014:405-435.

78. Antifungal azoles [for systemic use]. In: Aronson JK, ed. *Meyler's Side Effects of Drugs*. Sixteenth Edition ed. Oxford: Elsevier; 2016:584-601.

79. DrugBank. Sulfamerazine. https://go.drugbank.com/drugs/DB01581.

80. Padberg S. 2.6 - Anti-infective Agents. In: Schaefer C, Peters P, Miller RK, eds. Third Edition ed. San Diego: Academic Press; 2015:115-176.

81. Kuriyama T, Karasawa T, Williams DW. Chapter 13 - Antimicrobial Chemotherapy: Significance to Healthcare. In: Percival SL, Williams DW, Randle J, Cooper T, eds. *Biofilms in Infection Prevention and Control*. Boston: Academic Press; 2014:209-244.

82. Seley-Radtke KL, Yates MK. The evolution of nucleoside analogue antivirals: A review for chemists and non-chemists. Part 1: Early structural modifications to the nucleoside scaffold. *Antiviral Research.* 2018;154:66-86.

83. Farrell RE. Chapter 7 - Resilient Ribonucleases. In: Farrell RE, ed. *RNA Methodologies*. Fourth Edition ed. San Diego: Academic Press; 2010:155-172.

84. Shen Y, Li P, Chen X, et al. Activity of Sodium Lauryl Sulfate, Rhamnolipids, and N-Acetylcysteine against biofilms of five common pathogens. *Microbial Drug Resistance.* 2020;26(3):290-299.

85. Valaciclovir. In: Aronson JK, ed. *Meyler's Side Effects of Drugs*. Sixteenth Edition ed. Oxford: Elsevier; 2016:297-300.
