# Supplementary material for: Evaluation of SARS-CoV-2 entry, inflammation and new therapeutics in human lung tissue cells
Source: PLoS Pathog. 2022 Jan 13;18(1):e1010171. doi: 10.1371/journal.ppat.1010171 (PMC8791477; doi:10.1371/journal.ppat.1010171)
Supplement: S2 Text — (DOCX) [file ppat.1010171.s007.docx]

**Table S2. EC_50_ and CC_50_ of 39 antiviral drug candidates.**

|  | Vero E6 | HLT | | Vero E6 | HLT |  | | |  |
| --- | --- | --- | --- | --- | --- | --- | --- | --- | --- |
| Drugs | **EC_50_ (µM)** | | | **CC_50_ (µM)** | | | |  |  |
| Cepharanthine | 0.46 | | 6.08 | 22.37 | 16.15 | | **Concordant** | | |
| Luteolin | ~70.70 | | ~82.39 | >100 | >100 | |  |  |  |
| Ergoloid | 4.78 | | 9.17 | 18.67 | ~100 | |  |  |  |
| Ciclesonide | 20.52 | | 16.41 | >100 | >100 | |  |  |  |
| Licofelone | 87.50 | | 32.16 | >100 | ~53.61 | |  |  |  |
| Hydroxychloroquine | 1.58 | | 3.22 | >100 | ~100 | |  |  |  |
| Ivermectin | 13.94 | | 12.98 | 20.23 | ~100 | |  |  |  |
| Celecoxib | 12.99 | | 55.72 | 12.98 | ~100 | |  |  |  |
| Hypericin | 1.24 | | 0.31 | 4.80 | 0.14 | |  |  |  |
| Vidarabine | >100 | | 51.60 | >100 | >100 | |  |  |  |
| Eriodictyol | No effect | | 90.00 | >100 | >100 | | **Discordant** | | |
| Quercetin | >100 | | No effect | >100 | >100 | |  |  |  |
| Camostat | No effect | | 3.30 | >100 | >100 | |  |  |  |
| Phenformin | No effect | | 38.16 | >100 | >100 | |  |  |  |
| Valaciclovir | No effect | | >100 | >100 | >100 | |  |  |  |
| Sulindac | No effect | | >100 | >100 | >100 | | **No effect** | | |
| SLS | ~26.45 | | >100 | 87.05 | ~83.2 | |  |  |  |
| Myricetin | No effect | | No effect | >100 | >100 | |  |  |  |
| Sitagliptin | No effect | | No effect | >100 | >100 | |  |  |  |
| Dexamethasone | No effect | | No effect | >100 | >100 | |  |  |  |
| Pemirolast | No effect | | No effect | >100 | >100 | |  |  |  |
| Protilerin | No effect | | No effect | >100 | >100 | |  |  |  |
| Sulfamerazine | No effect | | No effect | >100 | >100 | |  |  |  |
| Carbazochrome | No effect | | No effect | >100 | >100 | |  |  |  |
| Higenamine | No effect | | No effect | >100 | >100 | |  |  |  |
| Tetrahydrobiopterin | No effect | | No effect | >100 | >100 | |  |  |  |
| Metformin | No effect | | No effect | >100 | >100 | |  |  |  |
| Vidagliptin | No effect | | No effect | >100 | >100 | |  |  |  |
| Monocaprin | No effect | | No effect | >100 | >100 | |  |  |  |
| Lauric acid | No effect | | No effect | >100 | >100 | |  |  |  |
| Monolaurin | No effect | | No effect | >100 | >100 | |  |  |  |
| Tazobactam | No effect | | No effect | >100 | >100 | |  |  |  |
| Nitrofurantoin | No effect | | No effect | >100 | >100 | |  |  |  |
| Cortisol | No effect | | No effect | >100 | >100 | |  |  |  |
| Ibuprofen | No effect | | No effect | >100 | >100 | |  |  |  |
| Indirubin | No effect | | No effect | >100 | >100 | |  |  |  |
| Glycyrrhizin | No effect | | No effect | >100 | >100 | |  |  |  |
| Sulfamethoxazole | No effect | | No effect | >100 | >100 | |  |  |  |
| Prednisone | No effect | | Some effect | >100 | >100 | |  |  |  |
