# Supplementary material for: Evaluation of SARS-CoV-2 entry, inflammation and new therapeutics in human lung tissue cells
Source: PLoS Pathog. 2022 Jan 13;18(1):e1010171. doi: 10.1371/journal.ppat.1010171 (PMC8791477; doi:10.1371/journal.ppat.1010171)
Supplement: Attachment — Submitted filename: Rebuttal letter.pdf [file ppat.1010171.s008.pdf]

Response to the Editor and Reviewers

Editor:

I have carefully read the manuscript and I concur with the opinion received from the reviewers. Specifically, below are the major criticisms following evaluation of this manuscript.

i) Considering this is a methodology based work and there are previous similar published work, the manuscript is considered not novel enough.

We concur with the editor that the study does not identify any new antiviral for SARS-CoV-2. Instead, using our model we confirm some entry inhibitors previously observed using other assays. However, we think that our study is novel in the following aspects: **i)** a significant percentage of selected drugs had discordant results between HLT and the conventional system VeroE6, **ii)** we report that IL-1b, but not interferons, modulates ACE2 expression in HLT cells, **iii)** camostat, cepharanthine, and ergoloid are three of the most potent drugs that inhibit SARS-CoV-2 entry, and importantly, also exert a significant anti-inflammatory effect on myeloid cells present in lungs, and **iv)** we show the suitability of the model to identify new entry factors. Thus, we believe our study highlights the need to use more physiological systems for the study of virus-host interactions and the identification of therapeutics, whereas adds new information not previously reported.

ii) Concerns were also raised about the limited sample size and limited reproducibility of the results given there are large variations in the results presented.

We have increased the sample size, which was n=2 donors, to an n=3-5 for each drug tested. In this sense, it is important to acknowledge that considering we tested 15 drugs in Figs 3 and 4, several donors were required to cover these screenings with suitable controls. In addition, we systematically perform experimental replicates for each condition, and results are highly reproducible with low variations in the majority of cases. Indeed, when we plot the EC_{50} values obtained for the individual experiments (New **Supplementary Figure 3E**), the results are very consistent and considered low for primary tissues. Thus, we show that our model is very reproducible.

iii) Insufficient information about the stability of the cultures/screening and lack of robust evaluation of the experimental system using WT SARS-CoV-2 or its variants.

We have performed new experiments to test the stability of the cultures during the length of the assay (both our virology and immunological assays were performed for 20h). Moreover, we have tested cell viability with and without cell infection and new data are presented in **Supplementary Figure 3C**. As expected for primary cell cultures, there is a slight decrease in the viability of most cell populations, which, however, does not preclude the performance of the assays. For example, as observed in the luciferase assays in **Figure 2D**, high numbers of RLU (median 22,000) are observed at 24h, and proper controls are included to ensure that low RLU values are not due to cell death. In this sense, we need to emphasize that a wide range of structural and immunological cell subsets are present at baseline in higher proportions, including the ones susceptible to infection, and is at baseline when we start our experiment (virological or immunological), being the readout at 20h. Still, we would like to highlight that the assay is always set up with fresh cells previous to any cell culture when cell viability is not compromised.

Additionally, and as suggested by the editor, we have validated our system using the SARS-CoV-2 delta variant. New data included in **Figure 4D** show that our model is suitable for screening entry inhibitors against different SARS-CoV-2 variants. Moreover, we have added additional donors in the experimental system using wild type virus (**Figure 4F**)

iv) Limitations of the model involving difficulty in obtaining human lung tissues and concerns about whether model truly represents lung infection model given high proportion of immune cells.

We have included a paragraph describing the potential limitations of the model, which reads as follows; *"limitations include the limited availability of human lung samples, inter-patient variation (age, smoking, etc.), the effects on lung biology of the medical condition instigating surgery, and the exact location of the sample resection, which may affect the proportion of cell subsets such as AT-II. However, this variability is what shapes the HLT into a more physiological and relevant model in comparison to current methods based on immortalized cell cultures".*

Regarding the high proportion of immune cells, we would like to highlight that the model was set up to obtain the highest number of both immune and epithelial cells. With this, we intended to be able to capture viral entry in any permissive cell (since lymphocytes or myeloid cells were originally proposed as potential targets), while we also aimed to have the interaction between the diverse subset populations coexisting in the alveolar parenchyma. We agree that modifications on the digestion protocol could lead to a higher number of non-immune cells. However, we think that the attractiveness of our model is its versatility to perform both the antiviral and the immune assays. Nevertheless, similar lung models have also shown high number of immune cells (Shuai Wang et al., Cell Research 2021; Matthew A. Schaller et al., JCI insight 2021). Importantly, our model is highly susceptible to SARS-CoV-2 infection and the presence of immune cells does not preclude the success of the viral entry or infection assays.

v) Inadequate consideration for TMPRSS2 and other SARS-CoV-2 entry determinants.

We have performed new analyses of AT-II enriched cells expressing ACE2 together with TMPRSS2 or with CD147 (**Supplementary Figure 3B**). Moreover, we have expanded the role of another entry factor and we have included new information, phenotypical and functional, regarding the expression of AXL in HLT cells, and their role in viral entry in these cells (**Supplementary Figure 3A** and **D**).

Reviewer #1: Grau-Expósito et al's manuscript "Rapid Evaluation of SARS-CoV-2 Entry, Inflammation and New Therapeutics in Human Lung Tissue Cells" described a translational model, HLT, for conronavirus studies. By using this model, the authors demonstrated the cell tropism of SARS-CoV-2 infection and test the efficacy of some potential drugs. Although this model is not perfect and some factors such as air-liquid interface might affect the conclusion drawn from their data, it still represents a useful bridge overcoming the gap between clinical outcome and cell line culture system/animal experiments. Therefore, I am pleased to endorse the publication of this manuscript in PLOS Pathogens.

<u>Major Issues: Key Experiments Required for Acceptance:</u> N/A

<u>Minor Issues: Editorial and Data Presentation Modifications:</u> N/A

Thank you very much for your positive response.

Reviewer #2: In this manuscript, Judith Grau-Expósito and colleagues developed a rapid platform to quickly evaluate the role of local inflammation in the expression profile of ACE2 and the entry of SARS-CoV-2, and identified novel anti-inflammatory compounds for COVID-19 treatment in the physiological model. In this model, the direct use of HLT cells, without long-term cell culture and in vitro differentiation approaches, preserves immune and structural cell populations and SARS-CoV-2 entry factors ACE2, CD147 and TMPRSS2 were also presented. Moreover, antiviral tests identified new compounds which were missed in conventional systems such as infection of VeroE6 cells. Using this method, they also showed that interferons do not modulate ACE2

expression, and stimulation of local inflammatory responses could be modulated by different compounds with antiviral activity.

Major Issues: Key Experiments Required for Acceptance:

The model in this study is of some interest because it preserves both structural cells and local immune cells in contrast to conventional screening systems such as infection of VeroE6. However, as a method-driven research, the novelty of the study is not enough. In addition, the model in the study is not a ready-to-use model as a rapid and simple evaluation system due to the limited availability of human lung tissues and lack of availability of experimental replicates and limited reproducibility. Further, the viability of human lung tissue cells in the absence or presence of SARS-CoV-2 infection were not described, which makes it hard to evaluate the application of the model. There are several specific concerns that also need to be addressed.

Specific points:

1. The biology properties of HLT cells depended on the donors. The authors need to compare the HLT cells from different donors. The heterogeneity of HLT from different donors would significantly impair the reproducibility and stability of the screening.
   We do agree with the reviewer that the availability of lung tissues is crucial for the success of this model. However, we have established a collaboration with the thoracic surgery department of the Vall d´Hebron Hospital for obtaining tissues regularly (2-3 samples/week). We think that this model could be easily set up in other medical research institutions, as long as there is a good collaboration between basic and clinical researchers. Regarding the experimental replicates, for each drug concentration, we always perform replicates, which are very consistent. Moreover, in the revised version of the manuscript, we have increased the number of donors for the antiviral testing in **Figure 3** and **4**. Now, each drug is tested in at least 3 different lung donors. Regarding the reproducibility, we have included a new graph showing the variability in the individual EC_{50} values obtained for each lung donor. We consider that our assay has very low variability for being a primary assay using tissue cells (**Supplementary Figure 3E)**.
   In the manuscript, however, we dedicate a paragraph to state the limitations of the study, which reads as *"limitations include the limited availability of human lung samples, inter-patient variation (age, smoking, etc.), the effects on lung biology of the medical condition instigating surgery, and the exact location of the sample resection, which may affect the proportion of cell subsets such as AT-II. However, this variability is what shapes the HLT into a more physiological and relevant model in comparison to current methods based on immortalized cell cultures".*

   Furthermore, we have compared cell subpopulations in lung tissues from n=15 donors. In new **Figure 1A** we observed that a similar proportion of main cell subsets are detected across different donors. Importantly, we only obtain samples from distal lung. Moreover, and as suggested by the reviewer, we have performed additional experiments testing cell viability with and without viral infection at baseline and at 20h (endpoint). We show that, in general, and as expected, cell viability is slightly lower after 20h of culture. Still, we would like to highlight that the assay is always set up with fresh cells previous to any cell culture when cell viability is not compromised. Thus, we believe that the slight loss in cell viability at 20h does not preclude the performance of the assay. This information has been included in the manuscript as a **Supplementary Figure 3C**.

2. The authors showed that human lung tissue cells in this model preserved local immune cells (CD45+) and structural cells (CD45-EpCAM+) (Figure 1). Thet defined CD45-EpCAM+ cells as AT-II like cells. However, EpCAM+ cells in lung tissue includes AT-I, AT-II as well as club cells. Therefore, the conclusions that ACE2 expression mainly in the putative AT-II cells (line145-146), 92.3%±1.14 of AT-II cells expressed CD147 (line151-

152) and TMPRSS2 expression profiles in AT-II cells (line153) are probably not accurate. More specific cell populations like HTII280+ and AQP5+ cells should be detected. In addition, gating of TMPRSS2+ACE2+ and TMPRSS2+CD147+ cells could be more suitable to identify susceptible cell populations of SARS-CoV-2 entry in this model. In addition, ACE2 is believed to be a functional receptor for SARS-CoV-2 virus entry. However, several publications showed that ACE2 is expressed at a low level and several co-receptors, such as AXL and CD147 were also identified. Did the authors look at the expression of AXL in the lungs? Is it possible that the authors knockout ACE2 in the HTL cells and then analyse cell infectivity?

We apologize for the confusion, but AT-II cells were not only defined as CD45-EpCAM+ cells, which would include all epithelial cells as the referee mentions. Instead, we systematically used the combination of CD45-CD31-EpCAM+ and HLA-DR+ markers to enrich for AT-II cells in the alveolar parenchyma. This was based on several references: Hasegawa et al, Respir Res 2017; Glanville et al, Am Rev Respir Dis 1989; Peters et al, Lab Invest 1990; Cunningham et al, J Cell Sci 1994; Cunningham et al, Immunology 1997; Corbiere et al, Respir Res 2011). Importantly, in the bronchial tissue, ciliated epithelial cells could also express HLA-DR, but in the alveolar parenchyma is mostly attributed to AT-II (Wosen et al, Front Immunol 2018).
To further confirm that this gate enriches for AT-II cells, we have performed new experiments and show that Surfactant Protein C (SPC), which is expressed exclusively by fully differentiated AT2 cells (Michael F. Beers, American Journal of Respiratory Cell and Molecular Biology 2017; Glasser, et al. J Aller Ther 2011) is restricted to this gate (New **Supplementary Figure 1B** and **Figure below**):

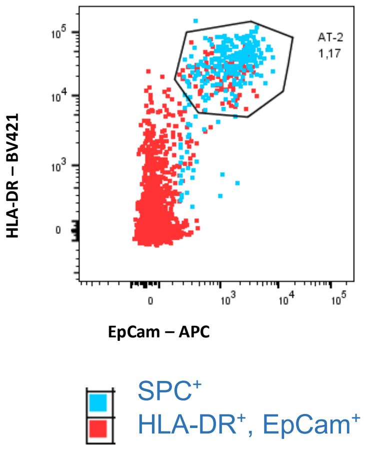

We have changed the wording to "*enriched AT-II*" (instead of AT-II like), in order to keep the percentages already reported relative to this gate. Of note, it was not compatible to test SPC and ACE-2 in the same flow cytometry panel.

Additionally, we have analyzed the proportion of double ACE2+CD147 and ACE2+TMPRSS2+ within the enriched AT-II fraction. The new data has been incorporated in **Supplementary Figure 3B**. We have also worked on the detection of the expression of AXL in HLT cells. We have successfully identified AXL+ cells in several cell populations. Importantly, the recombinant protein Axl is able to partially block the infection of SARS-CoV-2, indicating that the Axl protein also plays a role in viral entry, as previously suggested (Wang et al, Cell Research 2021). All this new data has been added in the **Supplementary Figures 3A** and **D**. Finally, in the previous version of the manuscript we

had already blocked ACE2 in HTL cells using anti-ACE2 antibodies, which showed that cell entry was diminished. These inhibition data is presented in **Figure 2F**.

3. In Figure 2, the VSV pseudovirus entry assay was performed in various donor-derived HLT cells. The statistical analysis was missing (Fig.2F). In addition, in lines 215-216, the authors mentioned that TMPRSS2 is not expressed in Vero E6 cells, so TMPRSS2 inhibition assay in Vero E6 cells could be used as a negative control in Fig. 2F.
The statistical analyses have been now incorporated in **Figure 2F**. As suggested, we have performed TMPRSS2 inhibition assays in VeroE6. Results are in line with the lack of TMPRSS2 expression in VeroE6, and have been added to **Figure 2B**. Also, the text has been modified accordingly (lines175-177).

4. In Figure 3, the authors identified some new antiviral compounds which were missed in Vero E6 cell screening. The results were reasonable because HLT cells are composed of multiple cell types in contrast to Vero E6 cells. Are there any specific target cell types identified in HLT cells after certain drug treatment? The identification of drug-targeted cell populations in HLT model is important since it could help the clinical transformation of these newly identified drugs.
We agree with the reviewer that the identification of specific cell targets after certain drug treatment would represent an important finding, however, this is difficult to demonstrate. Such experiments require using the GFP+ system in which the target is clearly the enriched AT-II fraction, as shown in **Figure 2E**. However, after effective drug treatment (i.e. camostat) infection is mostly abrogated and, thus, there are no longer detectable by GFP+. Consequently, cell targets cannot be properly identified.

5. In Figure 4, it seemed that newly identified drugs in HLT model showed weak inhibitory effect on SARS-CoV-2 replication and the statistical analysis is missing. In the Introduction, the authors mentioned that plitidepsin targeting the eEF1A host protein has shown potent preclinical efficacy against SARS-CoV-2 infection. Did the authors test plitidepsin in this model?
We agree with the reviewer that drugs do not show a strong inhibition in HLT cells. In this sense, camostat, which has been tested before in precision-cut lung slices from donors, was reported to be non-active at 0.5μM, and only showed approx. 40% inhibition at 10μM, whereas in entry assay it was highly effective (Hoffmann et al, EBiomedicine 2021). Similar results were observed by Hempel et al., (Chemical Science 2021), which observed 40% inhibition at 10μM for donor 1 and 60% inhibition for donor 2. These results are in agreement with our data. Moreover, we and others have observed that variability between technical replicates and donors is magnified in primary cultures when using replication-competent isolates. As suggested by the reviewer, we have added the statistics in **Figure 4F**, which shows statistically significant decreases in viral replication after drug treatments. Regarding the plitidepsin, we have not tested this drug in our system. We have set up the replication-competent virus assay for entry inhibitors testing, and the proper identification of drugs that inhibit other steps of the viral cycle might require an optimization of the protocol, which is out of the scope of the present study.

<u>Minor Issues: Editorial and Data Presentation Modifications</u>:

Some spelling and grammatical errors need to be corrected.

Thanks, we have proof-read the manuscript

Reviewer #3: In this study, Grau-Exposito et al, describe an interesting ex vivo culture system to examine SARS-CoV-2 infection in the lung. This system maintains many of the cell types that are important for infection and immunity in vivo. Using this system, they confirm SARS-CoV-2 pseudovirus entry and infection, and are able to test a number of entry inhibitors- showing similar

efficacy to other model systems. They also examine the effects of compounds on inflammatory responses in the mixed-cell population and determine that myeloid cell-induced inflammation may be modulated by certain drugs. The findings presented here are not novel, rather, the authors recapitulate previous findings as a proof-of-principle for their model.

Major Issues: Key Experiments Required for Acceptance:

1. The stability of these cultures is not clear. The authors present them as an alternative to differentiated epithelial cultures or organoids; however, the cells need to be freshly harvested for each experiment. Flow cytometric analysis of the populations is done at isolation, but several of the experiments require infection or treatment over the course of several days. It would be good to have further population analysis at these later timepoints to confirm that all cell types are present and that differences are not due to certain subtypes dying in prolonged culture.
   We thank the reviewer for this important point. We would like to highlight that we do not culture the cells for several days. Antiviral and immunological assays are performed within 20h from obtaining the fresh cell suspension. To this end, we have included viability data at 20h, with and without infection, which is shown in the new **Supplementary Figure 3C**.

2. The flow cytometric characterization of the cell populations in HLT cultures show much higher percentages of lymphocytes and neutrophils than AT-II or other epithelial cells. This raises the question of how well these cultures truly recapitulate the lung environment for SARS-CoV-2 infection. This becomes a particular concern in the level of inhibition of entry in HLT by hydroxychloroquine in Figure 3B. Entry in these AT-II cells should primarily depend on TMPRSS2 cleavage and therefore not be affected as much by HCQ treatment.
   We would like to highlight that we have optimized the cell isolation protocol for obtaining high numbers of both hematopoietic and epithelial cells. This could make our cell proportions slightly different than the data reported by methods optimized for epithelial cells only or for AT-II enrichment. Despite that, our results are quite consistent with other recent publications; high numbers of hematopoietic cells are also observed in lung parenchyma tissue (Shuai Wang et al., Cell Research 2021; Matthew A. Schaller et al., JCI insight 2021). Moreover, we have a substantial proportion of CD45 negative cells that were not phenotyped (i.e. fibroblasts) that are abundantly present in the lung parenchyma. This becomes more apparent in **Supplementary Figure 2**. However, and despite this low proportion of enriched AT-II cell targets, we successfully observed high and reproducible SARS-CoV-2 entry and replication in enriched AT-II cells.
   Regarding HCQ, we were also surprised to observe viral entry inhibition in HLT cells using HCQ for the reason mentioned by the reviewer. Indeed, we performed these assays with several different donors, and the results were extremely consistent. Note that similar results have been obtained using differentiated air-liquid interface cultures of proximal airway epithelium and 3D organoid cultures of alveolar epithelium (Mulay et al, Cell Reports 2021). However, multiple mechanisms of action have been postulated for HCQ, including interference in the endocytic pathway, blockade of sialic acid receptors and restriction of pH mediated S protein cleavage at the ACE2 binding site. A completed review can be found here (Satarker et al., Curr Pharmacol Rep 2020). This explanation has been added to the manuscript (lines 373-376).

3. Given the wide range of donor-based variability seen in most assays, especially those in Figures 5-6, using only 2 donors for the drug studies in Figures 3-4 seems inadequate.

   As suggested, we have increased the number of HLT donors in **Figure 3** and **4**. Now, each drug has been tested in at least 3 different lung donors.

Minor Issues: Editorial and Data Presentation Modifications:

1. Figure 1C- the label for endothelial cells is left off of this panel which makes the bar graphs accompanying the tSNE plots confusing.
   For the tSNE plots in the CD45- fraction, we focused on showing epithelial cells only to better display the expression of viral receptors within these fraction (for esthetical purposes).
2. Figure 2B, "anti-ACE Ab" should be changed to "anti-ACE2 Ab"
   This has been corrected.
